# Fractalkine/CX3CR1 Contributes to Endometriosis-Induced Neuropathic Pain and Mechanical Hypersensitivity in Rats

**DOI:** 10.3389/fncel.2018.00495

**Published:** 2018-12-21

**Authors:** Zhiming Liu, Sisi Chen, Chunping Qiu, Yaqiong Sun, Wenzhi Li, Jie Jiang, Jun-Ming Zhang

**Affiliations:** ^1^Department of Obstetrics and Gynecology, Qilu Hospital of Shandong University, Jinan, China; ^2^Key Laboratory of Gynecologic Oncology of Shandong Province, Qilu Hospital of Shandong University, Jinan, China; ^3^Pain Research Center, Department of Anesthesiology, University of Cincinnati College of Medicine, Cincinnati, OH, United States; ^4^Department of Obstetrics and Gynecology, Shandong Obstetrics and Gynecology Hospital, Jinan, China

**Keywords:** endometriosis, fractalkine, neuropathic pain, inflammation, microglia, peripheral hyperalgesia, central sensitization

## Abstract

Pain is the most severe and common symptom of endometriosis. Its underlying pathogenetic mechanism is poorly understood. Nerve sensitization is a particular research challenge, due to the limitations of general endometriosis models and sampling nerve tissue from patients. The chemokine fractalkine (FKN) has been demonstrated to play a key role in various forms of neuropathic pain, while its role in endometriotic pain is unknown. Our study was designed to explore the function of FKN in the development and maintenance of peripheral hyperalgesia and central sensitization in endometriosis using a novel endometriosis animal model developed in our laboratory. After modeling, behavioral tests were carried out and the optimal time for molecular changes was obtained. We extracted ectopic tissues and L4–6 spinal cords to detect peripheral and central roles for FKN, respectively. To assess morphologic characteristics of endometriosis-like lesions—as well as expression and location of FKN/CX3CR1—we performed H&E staining, immunostaining, and western blotting analyses. Furthermore, inhibition of FKN expression in the spinal cord was achieved by intrathecal administration of an FKN-neutralizing antibody to demonstrate its function. Our results showed that implanted autologous uterine tissue around the sciatic nerve induced endometriosis-like lesions and produced mechanical hyperalgesia and allodynia. FKN was highly expressed on macrophages, whereas its receptor CX3CR1 was overexpressed in the myelin sheath of sciatic nerve fibers. Overexpressed FKN was also observed in neurons. CX3CR1/pp38-MAPK was upregulated in activated microglia in the spinal dorsal horn. Intrathecal administration of FKN-neutralizing antibody not only reversed the established mechanical hyperalgesia and allodynia, but also inhibited the expression of CX3CR1/pp38-MAPK in activated microglia, which was essential for the persistence of central sensitization. We concluded that the FKN/CX3CR1 signaling pathway might be one of the mechanisms of peripheral hyperalgesia in endometriosis, which requires further studies. Spinal FKN is important for the development and maintenance of central sensitization in endometriosis, and it may further serve as a novel therapeutic target to relieve persistent pain associated with endometriosis.

## Introduction

Endometriosis is a common chronic inflammatory gynecologic disorder that is characterized by the growth of endometrial epithelial and stromal cells outside the uterus. It affects 6%–10% of women of reproductive age (Burney and Giudice, 2012; Laux-Biehlmann et al., 2015). The common symptoms of endometriosis are pain and subfertility (Brubel et al., 2017; Righarts et al., 2018). Pain symptoms associated with endometriosis include dysmenorrhea, dyspareunia, dysuria, dyschezia, and chronic pelvic pain, which profoundly affect quality of life (Simoens et al., 2012). Increasing evidences suggested that endometriotic pain is a type of inflammatory and neuropathic pain (Arosh et al., 2015; Ding et al., 2017; Possover, 2017). During ectopic endometrial growth, large numbers of macrophages, mastocytes, and neutrophils are recruited to participate in the inflammatory process (Laux-Biehlmann et al., 2015; Binda et al., 2017; Woo et al., 2017; Munros et al., 2018). Macrophages are the major source of pro-inflammatory cytokines/chemokines, including tumor necrosis factor-α (TNF-α), interleukin-1β (IL-1β), and monocyte chemoattractant protein 1 (MCP1; Cao et al., 2004; Sikora et al., 2018). The majority of these molecules were demonstrated to be involved in the pain pathogenesis (Mita et al., 2014; Neziri et al., 2014). Endometriotic lesion sites also exhibit increased neuroangiogenesis, which could be attributed to localized inflammation (Asante and Taylor, 2011; Arnold et al., 2012; Di Spiezio Sardo et al., 2015). Inflammatory molecules and neuropeptides might directly activate or sensitize sprouting sensory fibers in the tissues or excite nociceptive neurons, which resulted in pain and/or mechanical hypersensitivity (Liu et al., 2002; Mckinnon et al., 2015). Prolonged exposure of sensory neurons to inflammatory mediators would then lead to central sensitization (Arnold et al., 2012) and cause estrogen-independent pain (Woolf and Salter, 2000), as observed in some patients with endometriosis and persistent pelvic pain (Neziri et al., 2010; As-Sanie et al., 2013; Brawn et al., 2014).

Spinal microglia were thought to be the indicators of central sensitization in neuropathic pain (Watkins et al., 2001; Zhang et al., 2017; Ji et al., 2018; Lee et al., 2018; Tang et al., 2018). They were the first to become activated by peripheral nociceptive signals (Zhang et al., 2017), and could remain active for several weeks (Tanga et al., 2004; Clark et al., 2007a). Cytokines—including fractalkine (FKN)—were thought to be associated with microglia activation (Scholz and Woolf, 2007; Clark et al., 2012). FKN is a member of the chemokine family that consists principally of secreted proinflammatory molecules (Harrison et al., 1998), and acts by binding to a unique receptor, CX3CR1, expressed on corresponding cells (Bazan et al., 1997). The chemokines expressed in the periphery and central nervous system (CNS) have two forms: a membrane-bound protein and soluble forms (sFKN), each of which mediates different biologic functions (Bazan et al., 1997). In the periphery, predominant FKN-expressing cells are endothelial and epithelial cells. Membrane-bound FKN serves as an adhesion molecule to promote adhesion of leukocytes, while sFKN is a potent chemoattractant for the recruitment of leukocytes during chronic inflammation (Fong et al., 1998; Corcione et al., 2009; Schwarz et al., 2010). In the CNS, FKN-expressing cells are restricted to the intrinsic neurons and sensory afferents, whereas the FKN receptor (CX3CR1) is expressed exclusively in the spinal microglia (Verge et al., 2004; Clark et al., 2009; Yang et al., 2012). Similarly, elevated FKN was observed in the partial sciatic nerve ligation model of neuropathic pain, indicating a possible role for FKN in the development of pain (Zhuang et al., 2007; Clark et al., 2009).

Many clinical cases of endometriosis in the abdomino-pelvic cavity were reported to directly affect nerves (including the pudendal, obturator, femoral, and sciatic) or spinal roots (Possover and Chiantera, 2007; Possover et al., 2007, 2011; Possover, 2009; Manganaro et al., 2014). However, due to the limitations of animal models of pelvic endometriosis and difficulty in sampling nervous systems from patients with endometriosis, research in understanding the interactions between ectopic endometrium and nerves remains an intractable and challenging task (Chen et al., 2016). Given that increased levels of FKN were found in the peritoneal fluid of women with endometriosis (Ahn et al., 2015; still without a known role in endometriotic pain), herein we planned to explore the functional roles of FKN in contributing to the development and maintenance of peripheral hyperalgesia and central sensitization in endometriosis using a novel rat model of sciatic endometriosis that we originally described in a previous publication (Chen et al., 2016).

## Materials and Methods

### Animals

Adult female Sprague-Dawley rats (Huafukang Bioscience Co. Inc., Beijing, China) weighing approximately 220–280 g were housed in the Animal Experimental Center of Shandong University under controlled conditions (21–24°C, lights on 7:00–19:00 h). Food and water were provided *ad libitum*. The rat estrous cycle was not monitored since no marked effect on the estrous cycle was observed in the sciatic endometriosis rat model in previous studies (Chen et al., 2016). Carbon dioxide (100%) was used for rat euthanasia after the experiment or at the onset of emaciation, massive ascites, or CNS disorders. This study was approved by the Animal Ethics Committee of Qilu Hospital of Shandong University, Jinan, China.

### Establishment of a Rat Endometriosis Model

Rat endometriosis model was established as described previously (Chen et al., 2016), the uterus was exposed after a small midline abdominal incision was made under general anesthesia with 10% chloral hydrate (30 ml/kg per rat). A 1-cm segment of the right uterine horn was removed and temporarily kept in sterile phosphate-buffered saline (PBS) containing penicillin-streptomycin solution (100:1). The abdominal incision was sutured in layers with 4–0 silk. Rats were then repositioned on the left side, and a 1-cm incision was made at mid-thigh level to expose the sciatic nerve (under the fascia between the biceps femoris and quadriceps femoris muscles) on the right side. The sciatic nerve was then isolated by blunt dissection using a glass dissecting needle. A piece of uterine horn, approximately 5 mm in length, was cut from the prepared segment, wrapped around the sciatic nerve with the endometrium facing the nerve, and sutured in place using 6–0 silk. The incision was closed in layers after irrigation. For the Fat group, rats underwent a similar surgical procedure using fat tissue collected from around the uterus.

### Group Assignments and Specimen Collection

**Figure 1:**
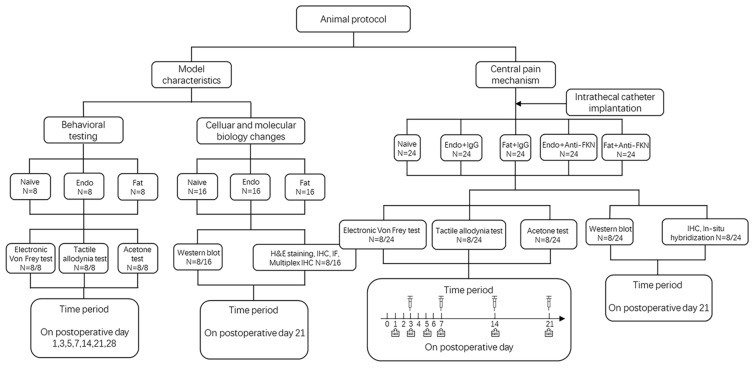
Flow diagram of the experiment. IHC, immunohistochemistry; IF, immunofluorescence.

Our study included two parts. The first part was to investigate pain pathogenesis and underlying molecular changes. The second part was to investigate central pain mechanisms. During the first part of the study, rats were randomly assigned into three groups with eight rats in each group: a Naïve group (no surgery); Endo group (with endometrial graft); and Fat group (with fat graft). Behavioral tests, western blotting, and H&E staining and immunohistochemistry (IHC) were performed to illustrate model characteristics and peripheral hyperalgesia. During the second part of our study we created five groups: a Naïve group (without surgery); Endo + IgG group (with endometrial graft and intrathecal injection of goat IgG); Fat + IgG group (with fat graft and intrathecal injection of goat IgG); Endo + Anti-FKN group (with endometrial graft and intrathecal injection of goat FKN-antibody); and Fat + Anti-FKN group (with fat graft and intrathecal injection of goat FKN-antibody). Behavioral tests (*N* = 8/per group); and western blotting (*N* = 8/per group), immunohistochemical, and hybridization analyses (*N* = 8/per group) were performed. On post-operative day (POD) 21, pain behaviors were demonstrated for each rat using electronic von Frey assessments before collecting samples. Then rats were deeply anesthetized and perfused transcardially with 4% paraformaldehyde after neutralizing antibody injection and behavioral testing; and the naïve and contralateral sciatic nerve, grafts, and L4-L6 levels of the spinal cord and dorsal root ganglia (DRG) were collected for H&E staining and IHC. Under deep anesthesia without perfusion fixation, the above tissues were removed quickly, placed into a tube, frozen, and stored in liquid nitrogen for western blotting analysis. A detailed protocol is shown in Figure 1.

### Intrathecal Catheter Implantation

Rats were anesthetized with 10% chloral hydrate, and a midline incision was made between L5 and L6 spinous processes at the level of the anterior superior iliac spines. A sterile polyethylene catheter-10 (PE-10, Smiths Medical) primed with sterile PBS was inserted through a small puncture in the intervertebral space between L5 and L6 spinous processes, and advanced cephalad about 2 cm to the lumbar enlargement of the spinal cord. The end of the tube was polished to avoid physical damage to the spinal cord. The lower back was sutured, and the exterior end was subcutaneously tunneled to exit through a small incision at the back of the neck. Rats showing neurologic deficits immediately after catheter implantation were excluded from subsequent administration or behavioral tests. Proper catheter placement was verified in each rat by injecting a small amount of lidocaine into the catheter after reviving the rat from anesthesia. Hind paw paralysis would indicate a successful catheterization. The precise placement of the catheter was checked in each rat by a postmortem examination of the spinal cord after the experiments were completed.

### Intrathecal Administration

To suppress FKN activity, 0.2 μg/μL FKN-neutralizing antibody (Clark et al., 2012; AF537, R&D) or vehicle (goat IgG, 5 μL, AB-108-C, R&D) was slowly injected through the intrathecal catheter on POD3–21 (time periods are shown in Figure 1). Each injection was followed by 20 μL flush with sterile PBS. The long-term anti-nociceptive effect of FKN-neutralizing antibody was assessed on POD 1, 3, 5, 7, 14, 21; and the acute effect of FKN-neutralizing antibody was assessed by electronic von Frey analysis at 0 h, 0.5 h, 1 h, and 3 h after injection on POD 3, 7, 14, 21.

### Outcome Measurements

All behavioral tests were measured in awake, unrestrained rats by the same experienced investigator blinded to group identity, as previously described (Chen et al., 2016). Prior to testing, all rats were habituated to the behavioral test set-up for several hours per day over 3 days and about 30 min immediately before each test.

#### Electronic Von Frey Test

Rats were tested for mechanical hypersensitivity using an electronic von Frey monofilament (CAMS & PUMC Institute of Biomedical Engineering, Tianjin, China) by measuring the threshold force required to cause foot withdrawal. Rats were tested for baseline thresholds before surgery.

#### Tactile Allodynia Test

A cotton wisp pulled from a Q-tip was swept gently across the plantar surface of the hind paw to determine the presence or absence of a brisk withdrawal response to a normally innocuous mechanical stimulation (light touch-induced tactile allodynia).

#### Acetone Test

Cold sensitivity was assessed by applying a drop of acetone (about 50 μL) onto the ventral surface of the hind paw to determine the presence or absence of a brisk withdrawal response to a normally innocent cold stimulation (cold allodynia).

#### Western Blotting Analysis

Tissues were homogenized in cold lysis solution (RIPA:PMSF:NaF:PPI, 100:1:1:1) and broken by ultrasonic cracking, then centrifuged at 12,000 rpm at 4°C for 20 min. The supernatants were collected and normalized for protein concentration. Proteins (80 μg) were subjected to 12% SDS-PAGE (sodium dodecyl sulfate-polyacrylamide gel electrophoresis), and then transferred onto a polyvinylidene fluoride membrane. The membranes were blocked at 37°C for 1 h in 5% BSA. After washing in TBS with 0.1% Tween 20, the membranes were incubated with goat anti-FKN (AF537, 1:400, R&D), rabbit anti-CX3CR1 (ab8021, 1:1,000, Abcam), rabbit anti–phosphorylated p38 (9211, 1:1,000, CST), or rabbit anti-p38 (8690, 1:1,000, CST), which were diluted in antibody dilution buffer (Beyotime, Shanghai, China) for 2 days at 4°C. After washing again, the membranes were incubated with secondary antibody for 1 h at room temperature. Bands were visualized through appropriate exposure to X-ray film (Kodak). Antibodies were subsequently eluted with stripping buffer and the membrane was incubated with rabbit anti-β-actin (4970; 1:1,000; CST) overnight at 4°C, followed by exposure the next day. Densitometric values of all the bands were measured with ImageJ software and normalized to the level of β-actin.

#### Immunohistochemistry

Tissues were embedded in paraffin after desiccation, and transverse paraffin sections were cut at a 4-μm thickness. After dewaxing, the sections were pre-treated for 20 min using sodium citrate buffer (pH = 6) for heat-mediated antigen retrieval. The sections were permeated with PBS containing 3% H_2_O_2_ for 10 min and blocked with 5% BSA for 30 min at 37°C. The sections were then incubated overnight with primary antibody diluted in PBS at 4°C followed by a PBS wash, and then incubation with secondary antibody for 1 h at 37°C. For IHC, primary antibodies included goat anti-FKN (AF537, 1:400, R&D) and rabbit anti-CX3CR1 (ab8021, 1:200, Abcam); and secondary antibodies included donkey anti-goat IgG (128625, 1:1,000, Jackson ImmunoResearch) and goat anti-rabbit/mouse IgG from immunohistochemical kits (SP9000, ZSGB-BIO). For fluorescent studies, primary antibodies included rabbit anti-FKN (ab25088, 1:100, Abcam), rabbit anti-CX3CR1 (ab8021, 1:100, Abcam), mouse anti-OX42 (MCA275R, 1:200, AbD Serotec; a marker for both quiescent and activated microglia), mouse anti-Iba1 (ab15690, 1:100, Abcam; a marker for activated macrophages/microglia), mouse anti-PGP9.5 (ab8189, 1:1,000, Abcam; a marker for myelinated and unmyelinated fibers), MBP (ab62631, 1:200, Abcam; a marker for the myelin sheath), and mouse anti-NeuN (ab104224, 1:1,000, Abcam; a neuronal marker). Secondary antibodies were goat anti-rabbit IgG Alexa Fluor 594 (ab150080, 1:200, Abcam) and goat anti-mouse IgG Alexa Fluor488 (ab150113, 1:200, Abcam). Both immunohistochemical and fluorescent images were captured with an Fluoview microscope (Olympus, Japan). For quantitative analysis, 4–6 sections per rat were sampled and used for calculations with Image-Pro^®^ Plus (Media Cybernetics, Rockville, MD, USA).

#### Opal-TSA for Multiplex Immunohistochemistry

Immunohistochemical analysis was used for each target (FKN, CX3CR1, Iba1, MBP) to optimize the antibodies and establish spectral libraries for multispectral analysis (Granier et al., 2017; Park et al., 2017; Parra et al., 2017). After dewaxing, antigen retrieval of paraffin-embedded sections (6-μm thickness) was performed by heating in Tris-EDTA buffer (pH9.0) for 15 min using microwave treatment (MWT). Sections were permeated with 3% H_2_O_2_ and then blocked with 5% BSA. The first primary antibody for MBP (ab62631, 1:2,000, Abcam) was incubated for 15 h at 4°C followed by HRP-labeled secondary antibody from an Opal™ 7-Color Manual IHC Kit (NEL811001KT, PerkinElmer). Visualization of MBP was accomplished using Opal 520 TSA Plus (dilution 1:50), after which the sections were placed in Tris-EDTA buffer (pH9.0) and heated using MWT. Then sections were incubated with primary antibody to Iba1 (ab15690, 1:250, Abcam) for 15 h at 4°C, followed by HRP-labeled secondary antibody from an Opal™ 7-Color Manual IHC Kit (NEL811001KT, PerkinElmer). Iba1 was then visualized using Opal 540 TSA Plus (1:50), and sections were placed in Tris-EDTA buffer (pH9.0) for MWT. Sections were then incubated with primary antibodies to CX3CR1 (ab8021, 1:1,000, Abcam) for 15 h at 4°C, followed by HRP-labeled secondary antibody from an Opal™ 7-Color Manual IHC Kit (NEL811001KT, PerkinElmer). CX3CR1 was visualized using Opal 620 TSA Plus (1:50), and sections were placed in Tris-EDTA buffer (pH9.0) for MWT. Sections were then incubated with primary antibodies to FKN (ab25088, 1:250, Abcam) for 15 h at 4°C, followed by HRP-labeled secondary antibody from an Opal™ 7-Color Manual IHC Kit (NEL811001KT, PerkinElmer). FKN was visualized using Opal 690 TSA Plus (1:50), and sections were placed in Tris-EDTA buffer (pH9.0) for MWT. DAPI was used for labeling nuclei. All images were captured and analyzed using a Vectra 3.0 Automated Quantitative Pathology Imaging System (PerkinElmer, Waltham, MD, USA).

#### *In situ* Hybridization

A CX3CR1 mRNA hybridization kit (MK3617, Boster) was used for hybridization analysis. After DRG sections (6 μm) were dewaxed and permeated with 3% H_2_O_2_, mRNA fragments were exposed with pepsin at 37°C for 20 min. A solution of 1% paraformaldehyde in 1/1,000 DEPC (diethyl pyrocarbonate) was then incubated for 10 min at room temperature. Prehybridization was performed at 40°C for 2 h, and CX3CR1 oligonucleotide probes (20 μL) were added to each section and covered with a glass coverslip for approximately 16 h at 40°C. Sections were washed twice with 2× standard saline citrate (SCC) for 5 min at 37°C, rinsed once with 0.5× SCC, and incubated twice with 0.2× SCC for 15 min at 37°C. After blocking, biotinylated rat anti-digoxin was incubated for 1 h at 37°C. After washing with PBS, sections were labeled with streptavidin-biotin complex for 20 min at 37°C. Biotin-peroxidase was added to sections for 20 min at 37°C, and they were finally dehydrated in a graded series of alcohols. Images were obtained with a microscope (Olympus, Japan) and analyzed with Image-Pro^®^ Plus (Media Cybernetics, Rockville, MD, USA).

#### H&E Staining

After dewaxing, sections were stained separately with hematoxylin and eosin solution (Solarbio, Beijing, China and ZSGB-BIO, Beijing, China) for 30 min at room temperature. Images were then captured under a light microscope (Olympus, Japan). Endometriosis-like lesions were confirmed by two gynecologic pathologists from Qilu Hospital of Shandong University.

### Statistical Analysis

Continuous data were presented as mean ± standard deviation. Categorical data were presented as percentage. All behavioral data were analyzed using 2-way repeated-measures analysis of variance (ANOVA). If ANOVA showed a significant interaction, Bonferroni’s multiple-comparison test was performed to determine the basis of the difference. For western blotting and IHC, data were analyzed by one-way ANOVA. Statistical analyses were performed and graphics were plotted using GraphPad Prism 5.0 software (GraphPad, La Jolla, CA, USA). The statistical software program IBM SPSS (version 22; Armonk, NY, USA) was used to analyze the correlations of expression levels for FKN/CX3CR1 in the sciatic nerve with severity of hyperalgesia. *p* < 0.05 was considered statistically significant.

## Results

### Ectopic Cysts That Form Around Adjacent Nerves Cause Inflammatory Cell Infiltration and Induce Mechanical Hypersensitivity and Cold Allodynia

**Figure 2:**
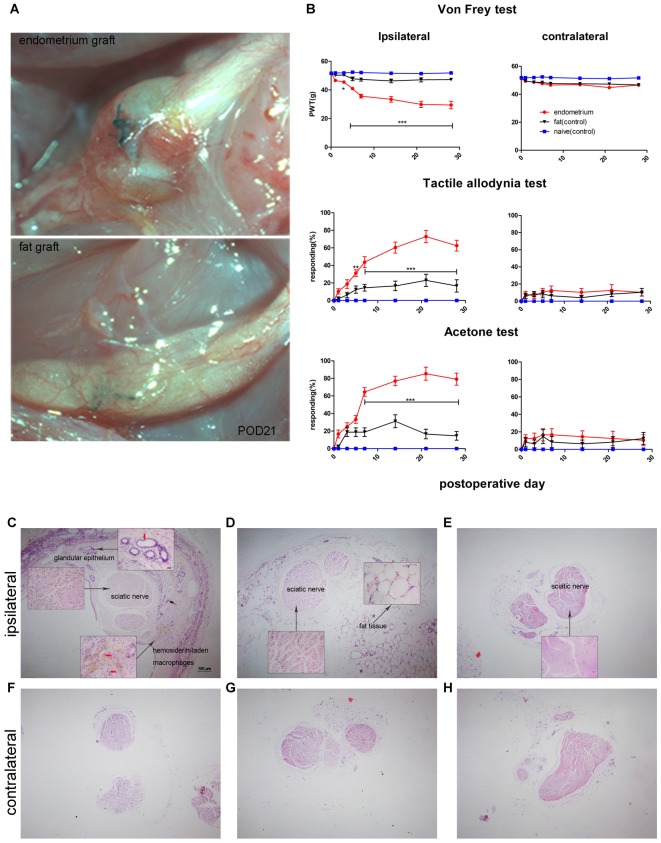
Rat sciatic endometriosis model. **(A)** Uterine grafts formed cysts around the sciatic nerve as visualized on postoperative day 21. **(B)** Behavioral changes in rats with sciatic endometriosis. Presurgical baseline values (mean of two test points) are presented as post-operative day (POD) 0. Mechanical hyperalgesia was determined by electronic von Frey filament. PWT, paw withdrawal threshold. Tactile allodynia and cold allodynia (acetone test) were measured by the percentage of withdrawal responses to a fine cotton wisp or a drop of acetone. In the uterine graft group, ipsilateral mechanical sensitivity and tactile allodynia were significantly different from the fat control group on all post-surgical days measured except for POD1. The ipsilateral cold allodynia response in the uterine graft group was significantly higher than the fat control group from POD7 to POD28. The mechanical sensitivity and allodynia of the contralateral hind paw showed no significant difference for any group. Two-way repeated-measures analysis of variance (ANOVA) was used for analysis *p < 0.05; **p < 0.01; ***p < 0.001. *N* = 8 rats per group. **(C)** H&E staining shows the ipsilateral sciatic nerve surrounded by endometrium, which exhibits typical glandular epithelium (vertical red arrow) and hemosiderin-laden macrophages (horizontal red arrows). Extensive inflammatory cells such as macrophages infiltrated into the nerve, and ectopic endometrium can bevisualized. **(D)** H&E staining shows the histologic structures in fat tissue with fat vacuoles. Fewer inflammatory cells can be seen in nervous and fat tissue. **(E)** Histologic features of the untreated sciatic nerve in naïve rats. Fewer inflammatory cells were observed. **(F)** Histologic features of the contralateral sciatic nerve with fewer inflammatory cells observed in the endometrial graft group. **(G)** Histologic features of the contralateral sciatic nerve in the fat graft group with fewer inflammatory cells observed. **(H)** Histologic features of the contralateral sciatic nerve in naïve rats without inflammatory cells observed. **(C–H)** 40×; insets, 400×. Scale bar for **(C–H)**, 100 μm; scale bar for insets, 20 μm. *N* = 8 rats per group.

As reported previously, ectopic endometrial tissue formed a single cyst filled with dark fluid that wrapped around the sciatic nerve in the uterine graft group (Figure 2A). Behavioral tests showed that rats with uterine grafts developed increased ipsilateral mechanical hypersensitivity and allodynia (including light touch-induced tactile allodynia and cold allodynia) compared to the contralateral hind paw and the control groups. Increased cutaneous sensitivity to mechanical and cold stimulation peaked at 7 days (*p* < 0.001) after initial surgery and was maintained throughout the testing period in the uterine graft group. During the peak of mechanical hypersensitivity and allodynia, the painful state was basically stabilized on POD21 (Figure 2B), and the nerve with uterine grafts or fat tissues was visually examined on this day. Further examination with H&E staining of the sciatic nerve cross-sections showed that uterine grafts exhibited typical glandular formations with columnar epithelial cells and hemosiderin-laden macrophages (Figure 2C). Fat tissue grafts only contained characteristic fat cells with clear vacuoles (Figure 2D). Large numbers of inflammatory cells including macrophages were observed in the sciatic nerve and adjacent uterine graft (Figure 2C), which were not shown in the contralateral hind paws and control groups (Figures 2E–H).

### Increased Expression of FKN and CX3CR1 in Ectopic Endometrium

**Figure 3:**
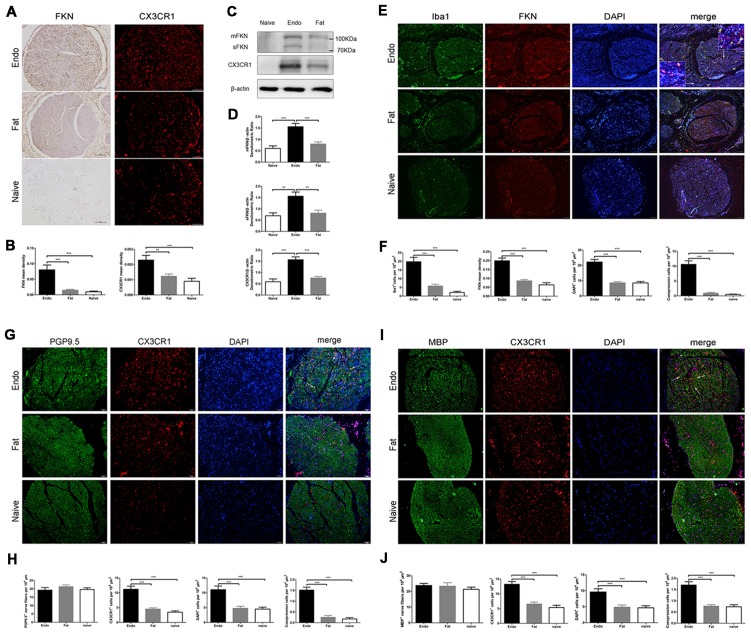
Expressions of fractalkine (FKN) and its receptor CX3CR1 in graft tissue. **(A)** Immunohistochemical staining for FKN and CX3CR1 in the sciatic nerve of graft tissue. **(B)** We analyzed FKN and CX3CR1 expression in the sciatic nerve of graft tissue by 1-way ANOVA. **(C)** Western blotting analysis showed protein levels of membrane-bound FKN, sFKN, and CX3CR1 in graft tissue. **(D)** Quantification of membrane-bound FKN, sFKN, and CX3CR1 bands in graft tissue, and analysis with 1-way ANOVA. **(E)** Immunofluorescence staining showed that FKN (red) was mainly expressed in macrophages labeled with Iba1 (green) in graft tissue. **(F)** The fluorescent images of FKN were statistically graphed for mean density, macrophage-positive cells, DAPI-positive cells, and co-expression in cells under 1-way ANOVA. **(G)** Immunofluorescent images show that CX3CR1 (red) was highly expressed in nerve fibers as indicated by PGP9.5 (green). The white arrows indicate the co-expression of CX3CR1 and PGP9.5 in cells. **(H)** Quantitative analysis of CX3CR1-positive cells, PGP9.5-positive cells, DAPI-positive cells, and co-expression in cells using 1-way ANOVA. **(I)** CX3CR1 was highly expressed on the myelin sheath when co-stained with MBP (green) using immunohistochemical staining. The white arrows indicate the co-expression of CX3CR1 and MBP in cells. **(J)** Quantitative analysis of CX3CR1-positive cells, MBP-positive cells, DAPI-positive cells, and co-expression in cells using 1-way ANOVA. *N* = 8 rats per group. **p < 0.01, ***p < 0.001. Arrows show co-expression. Scale bar for **(A,E,G,I)**, 100 μm. Insets, 5 μm.

**Figure 4:**
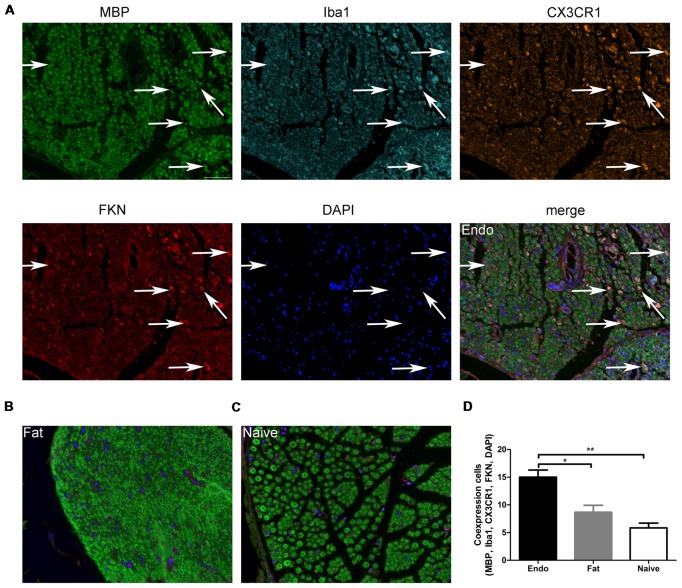
Myelin sheath, macrophages, and CX3CR1 and FKN expression in sciatic nerve. **(A)** Immunochemical staining for MBP, Iba1, CX3CR1, FKN, DAPI and the co-expression of these targets in the sciatic nerve of Endo group. **(B)** MBP, Iba1, CX3CR1, FKN, and DAPI co-expression in the sciatic nerve with fat graft group. **(C)** MBP, Iba1, CX3CR1, FKN, and DAPI co-expression in the sciatic nerve with Naïve group. **(D)** The number of positive cells co-expressing MBP, Iba1, CX3CR1, FKN, and DAPI was analyzed by 1-way ANOVA. *N* = 8 rats per group. *p < 0.05; **p < 0.01. Arrows show co-expression. Scale bar for **(A–C)**, 25 μm.

Here, we examined FKN/CX3CR1 expression in the ectopic lesions and compared it to fat and naïve control rats. Immunohistochemical staining revealed that both FKN and its receptor CX3CR1 were upregulated in the ectopic endometrium compared to normal endometrium and other graft tissues (Figures 3A,B, Supplementary Figures S1, S2). Expression of FKN/CX3CR1 in the ectopic lesions was negatively associated with pain threshold (FKN: *r* = −0.601 by Kendall test, *p* = 0.0001; *r* = −0.781 by Spearman test, *p* = 0.0001; CX3CR1: *r* = −0.558 by Kendall test, *p* = 0.0001; *r* = −0.735 by Spearman test, *p* = 0.0001), so positively associated with severity of hyperalgesia (Supplementary Figure S3). Western analyses were consistent with these results and further distinguished between membrane-bound FKN (~100 kDa) and sFKN (~80 kDa). The expression of sFKN was significantly increased in the endometriosis-like lesions (Figures 3C,D); and immunofluorescence images showed that there were significant numbers of macrophages infiltrating the uterine grafts identified with Iba1 (Figures 3E,F). To preliminarily determine whether FKN was from macrophages, double-immunostaining of FKN and Iba1 was examined, and this showed that membrane-bound FKN was located on the macrophages co-labeled with Iba1 in the ectopic endometrium; and that the co-expression was significantly greater than in the fat and naïve groups (*p* < 0.001 each; Figures 3E,F). In addition, CX3CR1 was overexpressed on the myelin sheaths of nerve fibers surrounded by uterine grafts, but was not observed in the fat and naïve groups where we attempted co-localization with PGP9.5 and MBP (Figures 3G–J). Multiplex immunohistochemical staining not only showed that the number of positive cells concurrently expressing FKN, CX3CR1, Iba1, and MBP were increased in the sciatic nerve of endo group; but demonstrated that macrophages were attached to the sciatic nerve via FKN/CX3CR1 binding (Figures 4A–D). These findings suggest that FKN/CX3CR1 contributes to peripheral hyperalgesia in endometriosis by mediating macrophage-nerve communication.

### Endometriosis-Induced Pain Is Associated With the Overexpression of FKN/CX3CR1/p38-MAPK and High Microglial Reactions in the Ipsilateral Spinal Dorsal Horn

**Figure 5:**
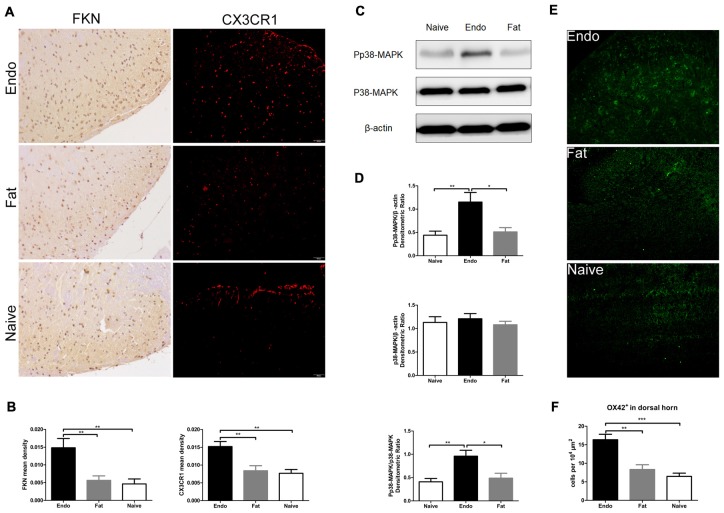
Increased expression of FKN/CX3CR1, phosphorylated p38-MAPK, and the number of microglia in the spinal cord of rats with sciatic endometriosis. **(A)** Immunohistochemical staining for FKN and CX3CR1 in the dorsal horn of L4–6 spinal cord. **(B)** FKN and CX3CR1 expression in the dorsal horn of L4–6 spinal cord was analyzed by 1-way ANOVA. **(C)** Western blotting experiments showing the protein levels of p38-MAPK phosphorylation in the L4–L6 spinal cord. **(D)** Quantification of p38-MAPK and pp38-MAPK bands in the L4–L6 spinal cord. **(E)** The total number of microglia in the dorsal horn was determined by counting OX42-positive cells. **(F)** Quantitative analysis of OX42-positive cells in the dorsal horn. One-way ANOVA was used for analysis. *N* = 8 rats per group. *p < 0.05; **p < 0.01; ***p < 0.001. Scale bar for **(A)**, 100 μm.

Since the sciatic nerve arising from the L4–6 DRG—as well as secondary pain neurons routed through the dorsal horn of the spinal cord—we evaluated the expression of FKN/CX3CR1 in the dorsal horn to investigate its role in central sensitization. IHC revealed that FKN/CX3CR1 was upregulated in the dorsal horn of L4–6 spinal cord compared to the fat graft or naïve rats (Figures 5A,B). Next, we examined by western blot analyses the phosphorylation of p38-MAPK in L4-L6 spinal cord, which was markedly increased in the uterine graft group compared with the fat graft or the naïve groups (Figures 5C,D). The total number of microglia in the dorsal horn was significantly higher than that in the control group as shown by the immunofluorescence staining (Figures 5E,F).

### Intrathecal Administration of FKN-Neutralizing Antibody Alleviates Endometriosis-Induced Pain and Hypersensitivity

**Figure 6:**
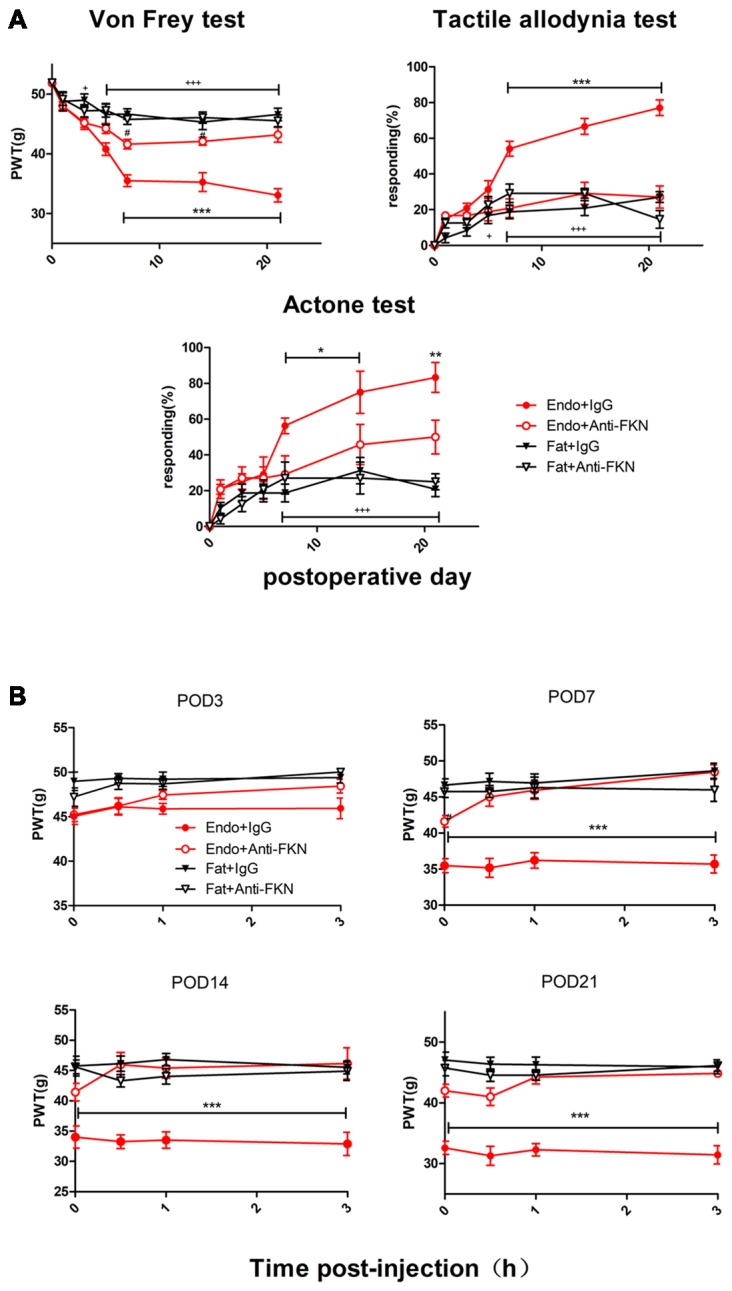
Enhanced mechanical hypersensitivity induced by endometriosis was reversed by intrathecal injection of FKN-neutralizing antibody. **(A)** Mechanical hyperalgesia, tactile allodynia, and cold allodynia were alleviated by FKN-neutralizing antibody. from POD7. **(B)** Endometriosis-associated pain behavior was not alleviated on POD3, but was reversed on POD7, 14, and 21. Two-way repeated-measures ANOVA was used for analysis. *N* = 8 rats per group. *p < 0.05; **p < 0.01; ***p < 0.001. *Represents the Endo + IgG group vs. Endo + Anti-FKN group. ^+^*p* < 0.05; ^+++^*p* < 0.001, ^+^represents the Endo + IgG group vs. Fat + IgG group. ^#^*p* < 0.05; ^#^represents Fat + Anti-FKN group vs. Endo + Anti-FKN group.

Endometriotic rats that received FKN-neutralizing antibody showed markedly reduced mechanical sensitivity, tactile allodynia, and cold allodynia between POD7 and POD21 (Figure 6A). We also examined acute effects of FKN-neutralizing antibody on different days after each injection. As shown in Figure 5B, the withdrawal thresholds remained unchanged on all testing days in the Fat + IgG, Fat + Anti-FKN, and Endo + Anti-FKN groups. In rats in the endometriosis plus FKN antibody group, however, the withdrawal threshold to mechanical stimulation began to return toward baseline on days 3, 7, 14, and 21 after injection. On POD3, the mechanical sensitivity of the Endo + Anti-FKN group was not significantly reversed immediately after injection compared with the Endo + IgG group. We also observed no statistically significant difference between the Endo + Anti-FKN and Fat + Anti-FKN groups after 1 h injection. These results suggested that the acute effects were present but not significant on POD3, probably because both pain and long-term effects of FKN-neutralizing antibody were not well developed at that time point. The acute effects remained significant until 3 h after the 0.5 h injection on POD7 and 0 h injection on POD 14 and 21, showing that the long-term effects might be well developed between POD7 and POD21; which is concordant with the results of the von Frey test shown in Figure 6A.

### Intrathecal Administration of FKN-Neutralizing Antibody Decreases FKN/CX3CR1 Expression in DRG

To estimate the effects of FKN/CX3CR1 in pain conduction, we tested their expression in L4–6 DRG. Immunohistochemical staining of DRG showed that FKN expression was primarily expressed in neuronal cells while intrathecal administration of FKN-neutralizing antibody decreased FKN expression in the Endo + Anti-FKN group. Soluble FKN was mainly observed in the nerve fibers around DRG neurons in the Endo + IgG group rather than other groups (Supplementary Figures S4A,C). *In situ* hybridization staining showed that CX3CR1 mRNA was overexpressed in the peri-neuronal cells in the Endo + IgG group while inhibition FKN also decreased CX3CR1 mRNA expression in DRG (Supplementary Figures S4B,D). These results suggest that FKN/CX3CR1 plays an important role in pain conduction in endometriotic rats.

### Intrathecal Administration of FKN-Neutralizing Antibody Attenuates p38-MAPK Activation, Decreases Microglial Signaling, and Downregulates CX3CR1 Expression in the Dorsal Horn

**Figure 7:**
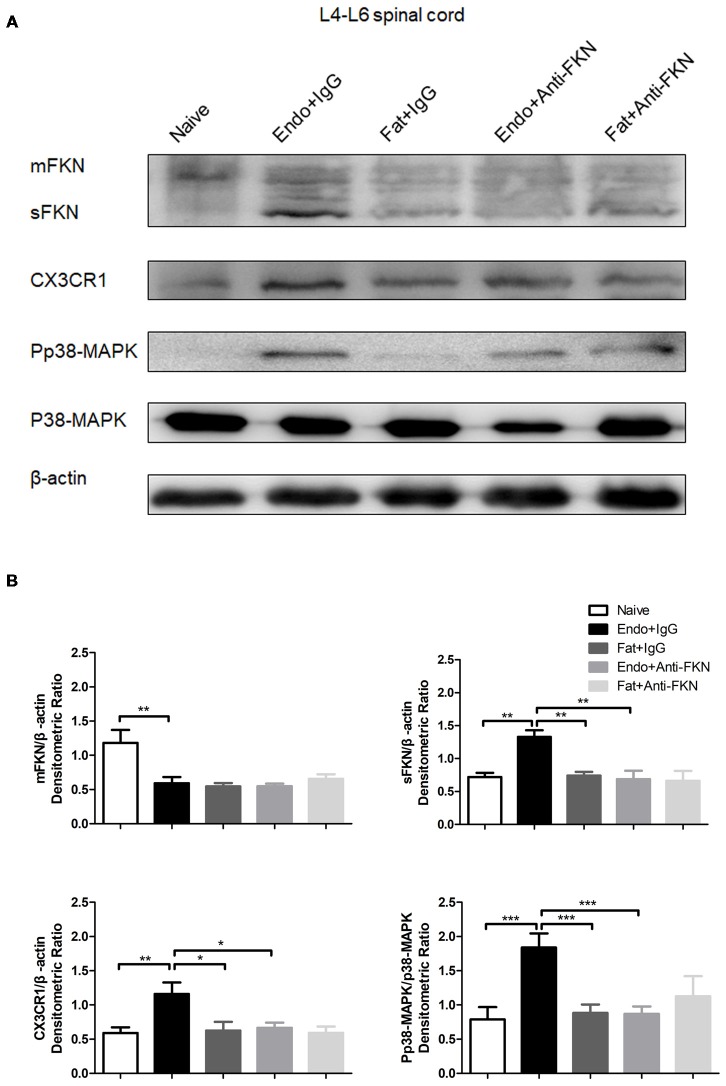
The expression of membrane-bound FKN, sFKN, CX3CR1, and pp38-MAPK in the spinal cord was significantly decreased by intrathecal administration of anti-FKN antibody on POD 21. **(A)** Western blot images showing protein levels of membrane-bound FKN, sFKN, CX3CR1, and pp38-MAPK in L4–6 spinal cord. **(B)** Quantification of membrane-bound FKN, sFKN, CX3CR1, and pp38-MAPK bands in the L4-L6 spinal cord. One-way ANOVA was used for analysis. *N* = 8 rats per group. *p < 0.05; **p < 0.01; ***p < 0.001.

The levels of pp38-MAPK and CX3CR1 expression were examined by the western blotting on POD 21 in endometriotic rats that received FKN-neutralizing antibody or control IgG. As expected, both pp38-MAPK and CX3CR1 were down regulated by blocking FKN. There was no significant difference among the naïve group, the Fat + IgG group, or the Fat + Anti-FKN group (Figures 7A,B).

**Figure 8:**
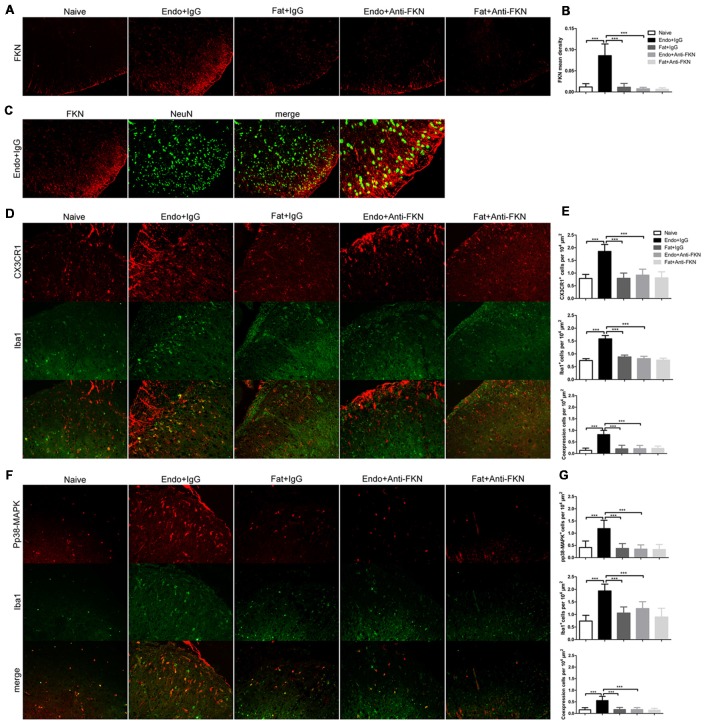
Effects of intrathecal administration of FKN-neutralizing antibody on spinal expression of FKN, CX3CR1, and pp38-MAPK as demonstrated by immunofluorescence staining. **(A)** Immunofluorescence staining showed expression of FKN in the dorsal horn. Intrathecal administration of FKN-neutralizing antibody markedly inhibited the expression of FKN. **(B)** Quantitative analysis of FKN mean density in the dorsal horn. **(C)** FKN (red) was co-expressed (yellow) with NeuN (green). **(D)** CX3CR1 expression in microglia was decreased in theEndo + Anti-FKN group. The number of activated microglia as determined by Iba1-positive cells was decreased by FKN-neutralizing antibody. **(E)** Quantitative analysis of CX3CR1-positive cells, Iba1-positive cells, and co-expression of CX3CR1 and Iba1 in dorsal horn cells. **(F)** Decreased expression of pp38-MAPK in microglia. **(G)** Quantitative analysis of pp38-MAPK-positive cells, Iba1-positive cells, and positive co-expression of pp38-MAPK and Iba1 in dorsal horn cells. One-way ANOVA was used for analysis. *N* = 8 rats per group. ***p < 0.001, *represents the Endo + IgG group vs. another group.

Effects of FKN-neutralizing antibody on FKN expression in the spinal cord were confirmed by immunofluorescence staining on POD 21 (Figures 8A,B). FKN in the spinal dorsal horn is mainly expressed by the neuronal cells as evidenced by co-labeling with NeuN, a known neuronal marker (Figure 8C). The activated number of microglia, as indicated by Iba1-positive staining and CX3CR1 expression in the microglia, were decreased in endometriotic rats receiving FKN-neutralizing antibody (Figures 8D,E). Similarly, expression of pp38-MAPK in microglia was reduced by treatment with neutralizing antibody on POD 21 (Figures 8F,G).

## Discussion

The sciatic endometriosis model was first described by our group in a recent publication and reproduced in the current study. The model was created by placing uterine tissue grafts around the sciatic nerve (Chen et al., 2016), and as shown in Figure 2A, a large cyst filled with dark fluid formed around the adjacent sciatic nerve on POD21. H&E staining revealed histopathologic characteristics of endometriosis and massive inflammatory cell infiltrations in the ectopic endometrium and sciatic nerve, similar to those endometriosis patients. Rats with endometrial grafts developed significant mechanical hypersensitivity and allodynia after approximately POD7.

The pain mechanisms underlying endometriosis are complex. Among these, inflammatory cytokines are an important initial factor, and increased sensory nerve fibers are the necessary final step in the pathogenesis of endometriotic pain. As we have previously reported (Chen et al., 2016), multiplex cytokine measurements from ectopic lesions showed that 10 cytokines containing FKN were up-regulated in endometriotic rats. FKN can bind directly to its single receptor CX3CR1, resulting in ligand-dependent changes in receptor function and biologic processes (Fong et al., 1998; Goda et al., 2000). Therefore, using our novel model we surmise that FKN/CX3CR1 might contribute to peripheral hyperalgesia in endometriotic pain.

In the present study, both membrane-bound and soluble forms of FKN as well as CX3CR1 were increased in the ectopic endometrial lesions, following with large numbers of macrophages infiltration. Combined with the positive correlation between FKN/CX3CR1 expression and severity of hyperalgesia, these results indicate that FKN/CX3CR1 expressed in the ectopic endometrial lesions was associated with peripheral hyperalgesia in endometriotic pain. Double fluorescent staining showed that membrane-bound FKN was primarily increased on macrophages, whereas CX3CR1 was expressed in the nerve fibers. Moreover, double-staining with MBP, a marker for myelin sheaths of nerve fibers, suggested that CX3CR1 was located on the Schwann cells that form the myelin, and this was supported by the lack of expression on DRG neurons (Verge et al., 2004). Multiplex staining further suggested that macrophages expressing membrane-bound FKN directly bind CX3CR1 anchored to myelin sheath of nerve fibers. It has been reported that, as a potent chemoattractant, soluble FKN attracts many inflammatory cells infiltration (Fong et al., 1998; Corcione et al., 2009; Schwarz et al., 2010), forming a stable inflammatory microenvironment around nerve fibers. Meanwhile membrane-bound FKN expressed on activated macrophages bind to CX3CR1 expressed on Schwann cells to trigger firm adhesion (Bazan et al., 1997; Fong et al., 1998; Mionnet et al., 2010; Schwarz et al., 2010; Doumas et al., 2015; Verheijden et al., 2015), which will cause not only local inflammation but myelin sheath degeneration and degradation through macrophage phagocytosis (Kotter et al., 2001; Chazaud et al., 2003; Campana et al., 2006; Martini et al., 2008; Klein and Martini, 2016; Jang et al., 2017). Moreover, the high-affinity binding also potentially activates Schwann cells by triggering intracellular receptor signaling and opening calcium channels (Imai et al., 1997; White et al., 2010; Chachi et al., 2013; Sheridan et al., 2014). Both the myelin phagocytosis and the activation of Schwann cells play a pivotal role in the onset of peripheral sensitization in peripheral ectopic lesions (Campana et al., 2006; Campana, 2007; Marinelli et al., 2012, 2014). Ours is the first report on the expression pattern of FKN/CX3CR1 signaling in ectopic endometrium, which contributes to peripheral hyperalgesia via macrophage-nerve interactions.

**Figure 9:**
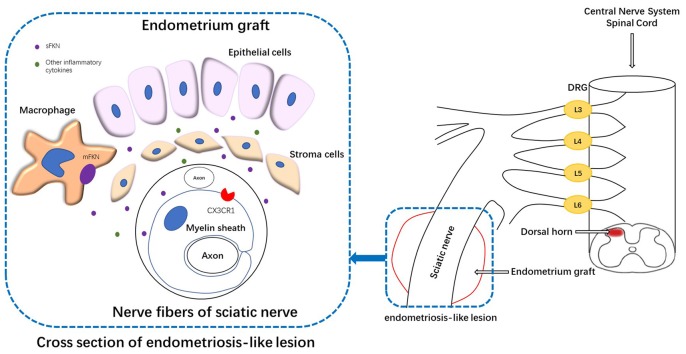
Schematic representation of the mechanisms underlying FKN release/action in endometriosis-like lesions. The endometriotic lesions contained a large number of inflammatory cells, including macrophages. The membrane-bound FKN expressed on macrophages may liberate sFKN, binding to its receptor CX3CR1, which is expressed on the myelin sheath of nerve fibers. FKN/CX3CR1 interaction represents a key regulatory mechanism for peripheral hyperalgesia. Nociceptive neuronal sensitization caused by FKN signaling in peripheral lesion reaches the spinal dorsal horn and traverses the DRG, inducing the expression of FKN/CX3CR1 and microglial activation within the dorsal horn; and ultimately develops into central sensitization. DRG, dorsal root ganglion.

Results of the present study indicated that the expression of FKN/CX3CR1/p38-MAPK and the number of microglia were significantly increased in the dorsal horn of endometriotic rats, which was concordant with the behavioral changes. Interestingly, the expression changes of FKN/CX3CR1 in the spinal cord was opposite to that in the peripheral nerves. FKN was expressed in neuronal cells while CX3CR1 was found in activated microglia as shown from other pain models (Clark et al., 2007b; Zhuang et al., 2007). It is likely that the FKN/CX3CR1 signaling in the ectopic endometrium causes sensitization of the nerve fibers through macrophage-nerve crosstalk, which may lead to altered mechanical hypersensitivity and allodynia. Conversely, sensory neuron activity generated from the ectopic lesion site (Chen et al., 2016) may cause overexpression of FKN from primary afferent neurons or spinal dorsal horn neurons and result in microglia activation via the CX3CR1/p38-MAPK pathway (Figure 9; Clark et al., 2007a; Xu et al., 2007; Crown et al., 2008); and this has been suggested to play roles in microglial activation and the persistence of neuropathic pain in models of peripheral nerve injury (Zhuang et al., 2007; Zhang et al., 2015). Enhanced microglial activation plays an essential role in the development of central sensitization (Clark et al., 2007a; Beazley-Long et al., 2018; Lee et al., 2018; Tang et al., 2018), which further exaggerates cutaneous hypersensitivity and pain. Thus, we speculate that endometriosis-induced persistent pain is associated with primary afferent neurons expressing FKN as well as FKN/CX3CR1/p38-MAPK pathway activation and enhanced microglial response in the dorsal horn.

DRG, as a kind of primary afferent neurons, plays a vital role in pain conduction. The expression of FKN in DRG neurons remained unaltered no matter whether operation, as other neuropathic pain models (Hughes et al., 2002; Verge et al., 2004; Clark et al., 2012). But soluble FKN was simply observed in endometriotic rats, possibly suggesting that ectopic lesions induce FKN release from DRG neurons. In turn, CX3CR1-positive cells such as microglia attracted by soluble FKN towards the area of the ongoing pathology (Verge et al., 2004) were overexpressed in rats with sciatic endometriosis, which also demonstrated the above point. On the other hand, inhibition FKN both decreased two forms of FKN and CX3CR1 expression in the DRG and alleviated pain behavior, further indicating that FKN/CX3CR1 signaling is relevant to endometriotic pain conduction.

Growing evidence supported the hypothesis that FKN in the spinal cord is important for microglial activation and neuropathic pain (Clark et al., 2012; Clark and Malcangio, 2014) in endometriotic rats. The role for spinal FKN/CX3CR1 in the development of endometriosis-administration of FKN antibody not only reversed the behavioral hypersensitivity that developed in rats with sciatic endometriosis, but also decreased the expression of FKN/CX3CR1 in the spinal cord. Repeated application of FKN antibody exhibited prolonged effects on behavioral sensitivity.

p38-MAPK was reported to be an important pain-related intracellular signaling pathway in neuropathic pain conditions (Xu et al., 2007; Crown et al., 2008). In the current study, pp38-MAPK in microglia was significantly increased in the spinal cord of endometriotic rats, and this was associated with behavioral changes. Blocking FKN in the spinal cord reduced the phosphorylation of p38-MAPK and the number of activated microglia. The CX3CR1/p38-MAPK pathway has been suggested to play roles in the microglial activation and the persistence of neuropathic pain in peripheral nerve injury models (Zhuang et al., 2007; Zhang et al., 2015). Results from the current study indicated that the FKN/CX3CR1/p38-MAPK pathway was equally important in the pain model of sciatic endometriosis.

In conclusion, results from the present study reveal that FKN released from a lesion site or expressed in the DRG and spinal dorsal horn plays different roles in the process of persistent pain and hypersensitivity associated with sciatic endometriosis. In the ectopic lesions, the cross-talk between recruited macrophages and myelin sheath of nerve fibers through FKN/CX3CR1 signaling cause a local inflammatory milieu and activate Schwann cells, which initiate the peripheral hyperalgesia. In the DRG and spinal dorsal horn, neuron-microglia communication takes a part in pain conduction and central sensitization in endometriosis, which was confirmed by intrathecal administration of FKN-neutralizing antibody and activated microglia. Despite that FKN/CX3CR1 signaling produces different effects in different cells in the development of endometriosis-induced pain, the way of this signaling in pain process is consistent, that is, ligand receptor binding to form cell-cell crosstalk. Thus, blocking FKN/CX3CR1 signaling communication in the different aspects of pain process can alleviate or reverse the development of pain symptoms in endometriosis. Findings from this study revealed a new mechanism underlying persistent pain associated with endometriosis by studying the interaction between inflammation and nerve, and might provide novel therapeutic targets for the management of this intractable clinical problem. Moreover, spinal FKN inhibition also offer reference materials for the treatment regimen on women with pelvic pain do not respond to therapies that are aimed at eliminating endometriotic lesions.

## Author Contributions

ZL, JJ, and J-MZ wrote the manuscript and were involved in all aspects of the experimental design. ZL largely performed the research procedures, including operations and behavioral assessments, as well as molecular tests and data analysis. SC, CQ, and J-MZ were responsible for experimental technology guidance and manuscript editing. YS and WL assisted with establishment of the rat model and with animal experiments. All authors have read and approved the final manuscript.

## Conflict of Interest Statement

The authors declare that the research was conducted in the absence of any commercial or financial relationships that could be construed as a potential conflict of interest.

## Supplementary Material

FIGURE S1 Expression of FKN, CX3CR1, and Iba1 in normal endometrium and endometriotic tissues. **(A)** Immunohistochemical staining for FKN, CX3CR1, and Iba1 in normal endometrium and endometriotic tissues. **(B)** FKN, CX3CR1, and Iba1 expression in normal endometrium and endometriotic tissues was analyzed using *t*-test. *N* = 8 rats per group. ***p* < 0.01. Scale bar for **(A)**, 50 μm, 100×.

FIGURE S2 Expression of CX3CR1 in the sciatic nerve of graft tissue. **(A)** Immunohistochemical staining for CX3CR1 expression in the sciatic nerve of graft tissue. **(B)** Quantitative analysis of CX3CR1 expression using 1-way ANOVA. *N* = 8 rats per group. ****p* < 0.001. Scale bar for **(A)**, 100 μm, 100×.

FIGURE S3 Correlation between FKN/CX3CR1 expression in the sciatic nerve of graft tissue and severity of hyperalgesia on POD21. **(A)** The relationship between FKN mean density in immunohistochemical staining and hind PWT obtained by an electronic von Frey test. A significant correlation was observed between these values by Kendall and Spearman correlation coefficients (*r* = −0.601 by Kendall test, *p* = 0.0001; *r* = −0.781 by Spearman test, *p* = 0.0001). **(B)** The relationship between CX3CR1 mean density in immunohistochemical staining and hind PWT obtained by electronic von Frey test. A significant correlation was observed between these values by the Kendall and Spearman correlation coefficients (*r* = −0.558 by Kendall test, *p* = 0.0001; *r* = −0.735 by Spearman test, *p* = 0.0001).

FIGURE S4 Expression of FKN and its cognate receptor CX3CR1 in L4–6 DRG. **(A)** Immunohistochemical staining showed stronger positive expression for FKN in neuronal cell bodies in the Naïve, Endo + IgG, and Fat + IgG group. Insets show expression of FKN (arrows) in the nerve fibers around a DRG neuron. **(B)** CX3CR1 mRNA was mainly observed over peri-neuronal cells in the Endo + IgG group. **(C)** We analyzed FKN expression in DRG neurons by 1-way ANOVA. **(D)** Quantification of CX3CR1mRNA expression in DRG, and analysis with 1-way ANOVA. *N* = 8 rats per group. ***p* < 0.01; ****p* < 0.001. **(A)** and insets, 600×; **(B)**, 400×. Scale bar for **(A)**, 10 μm. Insets, 10 μm. Scale bar for **(B)**, 25 μm.
