# Supplementary figures and images for: Fractalkine/CX3CR1 Contributes to Endometriosis-Induced Neuropathic Pain and Mechanical Hypersensitivity in Rats
Source: Front Cell Neurosci. 2018 Dec 21;12:495. doi: 10.3389/fncel.2018.00495 (PMC6309014; doi:10.3389/fncel.2018.00495)

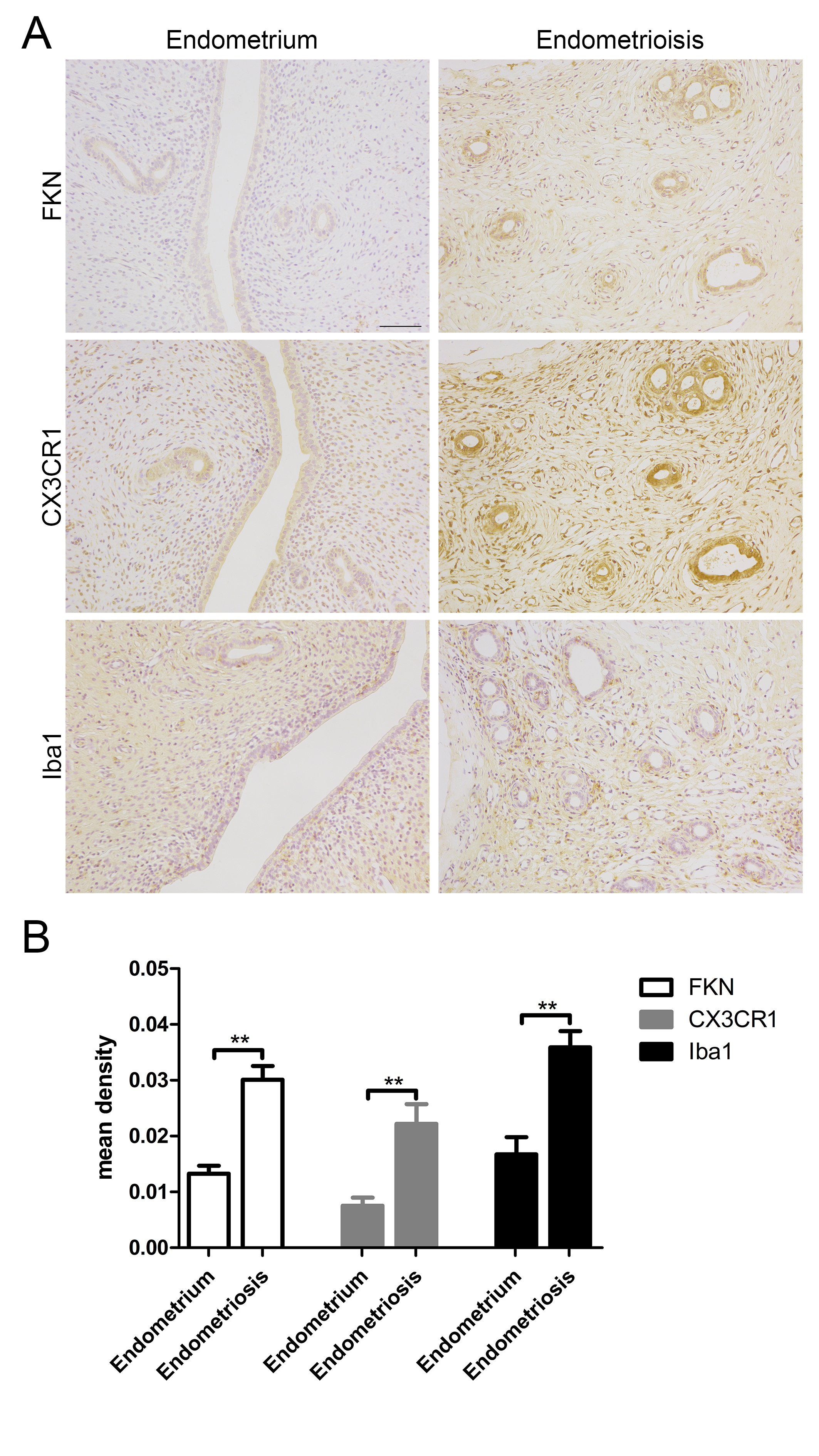

Supplement: FIGURE S1 — Expression of FKN, CX3CR1, and Iba1 in normal endometrium and endometriotic tissues. (A) Immunohistochemical staining for FKN, CX3CR1, and Iba1 in normal endometrium and endometriotic tissues. (B) FKN, CX3CR1, and Iba1 expression in normal endometrium and endometriotic tissues was analyzed using t-test. N = 8 rats per group. **p < 0.01. Scale bar for (A), 50 μm, 100×. [file Image_1.JPEG]

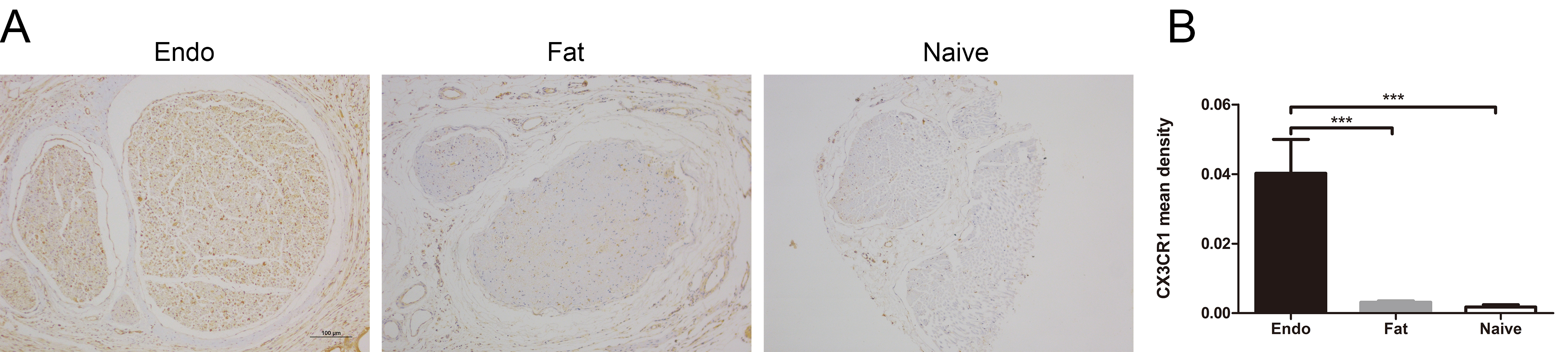

Supplement: FIGURE S2 — Expression of CX3CR1 in the sciatic nerve of graft tissue. (A) Immunohistochemical staining for CX3CR1 expression in the sciatic nerve of graft tissue. (B) Quantitative analysis of CX3CR1 expression using 1-way ANOVA. N = 8 rats per group. ***p < 0.001. Scale bar for (A), 100 μm, 100×. [file Image_2.JPEG]

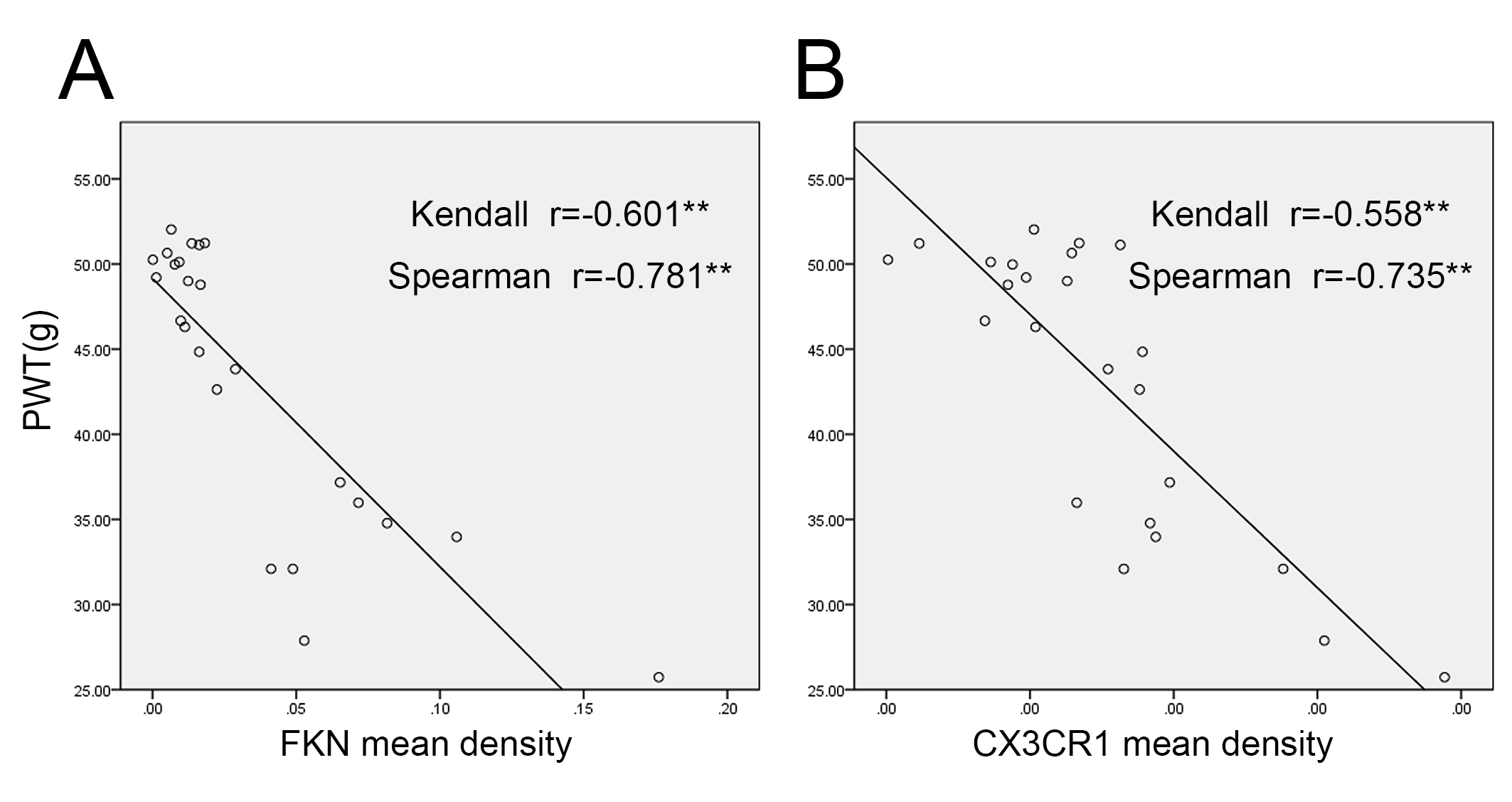

Supplement: FIGURE S3 — Correlation between FKN/CX3CR1 expression in the sciatic nerve of graft tissue and severity of hyperalgesia on POD21. (A) The relationship between FKN mean density in immunohistochemical staining and hind PWT obtained by an electronic von Frey test. A significant correlation was observed between these values by Kendall and Spearman correlation coefficients (r = −0.601 by Kendall test, p = 0.0001; r = −0.781 by Spearman test, p = 0.0001). (B) The relationship between CX3CR1 mean density in immunohistochemical staining and hind PWT obtained by electronic von Frey test. A significant correlation was observed between these values by the Kendall and Spearman correlation coefficients (r = −0.558 by Kendall test, p = 0.0001; r = −0.735 by Spearman test, p = 0.0001). [file Image_3.JPEG]

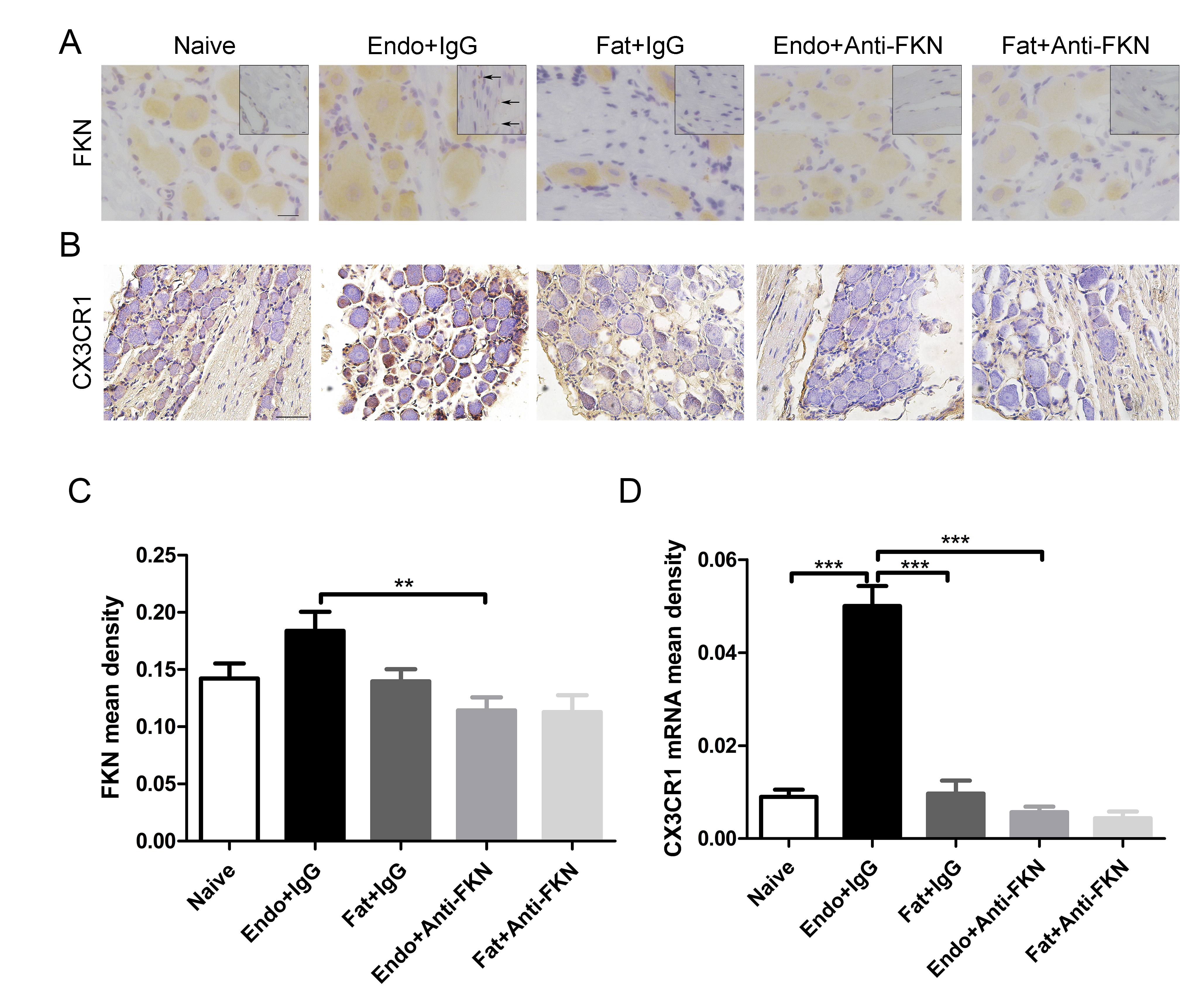

Supplement: FIGURE S4 — Expression of FKN and its cognate receptor CX3CR1 in L4–6 DRG. (A) Immunohistochemical staining showed stronger positive expression for FKN in neuronal cell bodies in the Naïve, Endo + IgG, and Fat + IgG group. Insets show expression of FKN (arrows) in the nerve fibers around a DRG neuron. (B) CX3CR1 mRNA was mainly observed over peri-neuronal cells in the Endo + IgG group. (C) We analyzed FKN expression in DRG neurons by 1-way ANOVA. (D) Quantification of CX3CR1mRNA expression in DRG, and analysis with 1-way ANOVA. N = 8 rats per group. **p < 0.01; ***p < 0.001. (A) and insets, 600×; (B), 400×. Scale bar for (A), 10 μm. Insets, 10 μm. Scale bar for (B), 25 μm. [file Image_4.JPEG]
